# Innovative Approach to Fast Electron Microscopy Using the Example of a Culture of Virus-Infected Cells: An Application to SARS-CoV-2

**DOI:** 10.3390/microorganisms9061194

**Published:** 2021-05-31

**Authors:** Marion Le Bideau, Nathalie Wurtz, Jean-Pierre Baudoin, Bernard La Scola

**Affiliations:** 1 Microbes, Evolution, Phylogeny and Infection (MEPHI), UM63, Institut de Recherche pour le Développement (IRD), Assistance Publique—Hôpitaux de Marseille (AP-HM), Aix-Marseille University, 13005 Marseille, France; Marion.LE-BIDEAU@ap-hm.fr (M.L.B.); nathalie.wurtz@univ-amu.fr (N.W.); 2 IHU Méditerranée Infection, 13005 Marseille, France

**Keywords:** SARS-CoV-2, electron microscopy, embedding method, microwaves, single-break strip

## Abstract

Despite the development of new diagnostic methods, co-culture, based on sample inoculation of cell monolayers coupled with electron microscopy (EM) observation, remains the gold standard in virology. Indeed, co-culture allows for the study of cell morphology (infected and not infected), the ultrastructure of the inoculated virus, and the different steps of the virus infectious cycle. Most EM methods for studying virus cycles are applied after infected cells are produced in large quantities and detached to obtain a pellet. Here, cell culture was performed in sterilized, collagen-coated single-break strip wells. After one day in culture, cells were infected with SARS-CoV-2. Wells of interest were fixed at different time points, from 2 to 36 h post-infection. Microwave-assisted resin embedding was accomplished directly in the wells in 4 h. Finally, ultra-thin sections were cut directly through the infected-cell monolayers. Our methodology requires, in total, less than four days for preparing and observing cells. Furthermore, by observing undetached infected cell monolayers, we were able to observe new ultrastructural findings, such as cell–cell interactions and baso-apical cellular organization related to the virus infectious cycle. Our innovative methodology thus not only saves time for preparation but also adds precision and new knowledge about viral infection, as shown here for SARS-CoV-2.

## 1. Introduction

Electron microscopy (EM) in biology is a powerful tool for examining the morphology and ultrastructure of cells. In the virology field, EM examination of viruses grown in cultured cells has been a key tool in determining the etiologic agent in numerous disease outbreaks caused by previously unknown viruses and remains the gold standard for understanding cellular virus infectious cycles [1,2,3,4,5]. While EM aimed at identifying viruses based on their morphology can be performed on small samples, such as cell culture supernatants or patients samples, the different steps of the viral life cycle (attachment, entry, replication, assembly, and egress) and the ultrastructure of the infected cell are studied by EM examination of virus-infected cultured cells [6,7,8].

One standard methodology that we have been extensively using in our laboratory for accessing the ultrastructure of virus-infected cultured cells consists in production of a pellet of infected cells after mechanical or trypsin detachment of the cells from the substrate, chemical fixation or cryo-fixation of the cells, and then resin embedding of the cell pellet [7,9]. Ultramicrotomy is then performed on the pellet and observations are made on heavy metal contrasted ultra-thin sections by transmission or scanning electron microscopy. Overall, this approach has proven time-consuming (~three weeks), from virus-infected cells production by sequential sub-cultures to EM processing and imaging, and provided images of detached, pelleted cells, with fragile cells at the most advanced stage of infection typically bursting during preparation.

Infected cultured cells can also be prepared for EM as monolayers via flat embedding. Cell monolayers can be grown on flat supports, such as glass or plastic coverslips, sapphire discs, EM grids, and nitrocellulose filters [7,10]. Flat embedding of cell monolayers combined with plane sectioning of the cells, with the cutting plane parallel to the substrate, has proven a powerful method for accessing information about the ultrastructure of cells grown in two dimensions (2D), especially migrating cells [11]. In the field of virology, flat embedding and plane sectioning of virus infected cells has been used in only a few studies, for example with cells grown on ACLAR embedding films [12] or with cells grown on gridded slides for correlative light–electron microscopy studies [13].

Here, we developed a novel approach for EM after in-well flat embedding and plane sectioning of virus-infected cells, using breakable cell culture dishes and microwave EM processing. Our approach can be broadly used for fast infectious cycle examination by EM, providing images of undetached cell monolayers at time points selected by light microscopy during a single cell culture round. We show here its successful use for studying the infectious cycle of SARS-CoV-2 in VeroE6 cell monolayers.

## 2. Materials and Methods

### 2.1. Cell Culture and the Virus Infectious Cycle

Substrate preparation was performed by Cell and Soft (Grenoble, France). Briefly, Greiner Bio-One 96 well single-break strip microplates (Greiner Bio-One, Frickenhausen, Germany) were UV sterilized at 365 nm (125 mW/cm^2^) for 5 min (UV-KUB 1, Kloé, France) under a laminar flow hood. Collagen coating was then performed under a sterile laminar flow cabinet. Subsequently, 400 ng of rat tail collagen I (Corning life science, Amsterdam, The Netherlands) was added to each well and incubated at 37 °C overnight. Plates were stored at 4 °C until used at our laboratory. Vero E6 cells (ATCC^®^ CRL-1586^™^) were suspended at 2 × 10^5^ cells/mL in MEM supplemented with 10% fetal calf serum and 1% L-glutamine (M10 medium). Then, 200 μL of this cell suspension was distributed into each modular well of two removable single-break strips (8 wells each) in a microplate and incubated for 24 h at 37 °C with 5% CO_2_. Vero E6 cell monolayers were infected in one of the two strips by removing culture medium and adding 50 µL of SARS-CoV-2 viral suspension (IHUMI-3 strain) (MOI of 0.1) in the MEM medium supplemented with 4% fetal calf serum and 1% L-glutamine (M4 medium). The IHUMI3 strain was the third isolated in our laboratory form a nasopharyngeal swab in March 2020 under previously described conditions [14]. The second strip was used as a negative control by adding 50 μL of the M4 medium only and both strips were centrifuged for 1 h at 37 °C at 2272× *g*. The supernatant from the wells was discarded, the cells rinsed gently three times with M4 medium and 200 µL of M4 medium was added to the 8 wells. Cells were incubated at 37 °C and 5% CO_2_. For each post-infection time point (2, 3, 6, 12, 18, and 36 h), one infected and one non-infected modular well was fixed by adding 30 μL of 25% glutaraldehyde in 0.1 M sodium cacodylate buffer and stored at 4 °C until infectious cycle completion.

### 2.2. Electron Microscopy

Cells were fixed with glutaraldehyde (2.5%) in 0.1 M sodium cacodylate buffer. Resin embedding was microwave-assisted with a PELCO BiowavePro+ (Ted Pella Inc., Redding, CA, USA; Eloïse, France) (Table S1), by exchanging 200 µL of the different solutions at each step. Samples were washed two times with a mixture of 0.2 M saccharose/0.1 M sodium cacodylate and post-fixed with 1% OsO4 diluted in 0.2 M potassium hexa-cyanoferrate (III)/0.1 M sodium cacodylate buffer. After two washes with distilled water, samples were gradually dehydrated by successive baths in 30%, 50%, 70%, 90%, 96%, and 100% ethanol. Substitution with Epon resin (Embed 812 mixed with NMA, DDSA, and DMP-30 hardener; Electron Microscopy Sciences, Hatfield, PA, USA) was achieved by incubations with 25%, 50%, 75% Epon resin in ethanol and incubations with 100% Epon resin. A final volume of 275 µL of 100% Epon resin was added to each well, and prior to microwave-curing, a 3D-printed sealing cap was placed on top of the wells to protect the resin from water in the microwave polymerization chamber. Instructions for preparing sealing caps are available at the link below. Sealing caps were designed by using Autodesk Fusion 360 software. The cap file created was exported as an stl extension file and reworked with Simplify 3D software to enter 3D print settings. The sealing caps were manufactured with Python Flex, 1.75 mm diameter, a flexible thermoplastic polyurethane (TPU) filament (Form futura, Nijmegen, The Netherlands). On the Simplify 3D software, the standard properties of the Ninjaflex filament were selected: 25% filling rate, addition of a skirt composed of 3 layers and rapid printing quality (200 µm). The 3D printer used to make this cap was the 30 Pro MK2 volumic stream printer (Volumic, Nice, France). Printing was carried out continuously, layer by layer. The G-Code file for this cap can be downloaded at [15]. Resin microwave-curing was performed for a total of 2 h. All solutions used above were 0.2 µm filtered. After curing, the resin blocks were manually trimmed with a razor blade and dish bottoms were detached from cell monolayers by cold shock via immersion in liquid nitrogen for 20 s and removal of the plastic with pliers. Resin blocks were placed in a UC7 ultramicrotome (Leica Biosystems, Wetzlar, Germany), trimmed to pyramids, and ultrathin 70 nm sections were cut and placed on HR25 300 Mesh Copper/Rhodium grids (TAAB, Reading, UK). Sections were contrasted according to Reynolds [16]. Grids were attached to a double-side tape on a glass slide and platinum-coated at 10 mA for 20 s with a MC1000 sputter coater (Hitachi). Electron micrographs were obtained on a SU5000 SEM (Hitachi High-Technologies, HHT, Japan) operated in high-vacuum at 7 kV accelerating voltage and observation mode (spot size 30) with BSE detector and magnifications ranging from ×1500 to ×40,000.

## 3. Results

**Figure 1:**
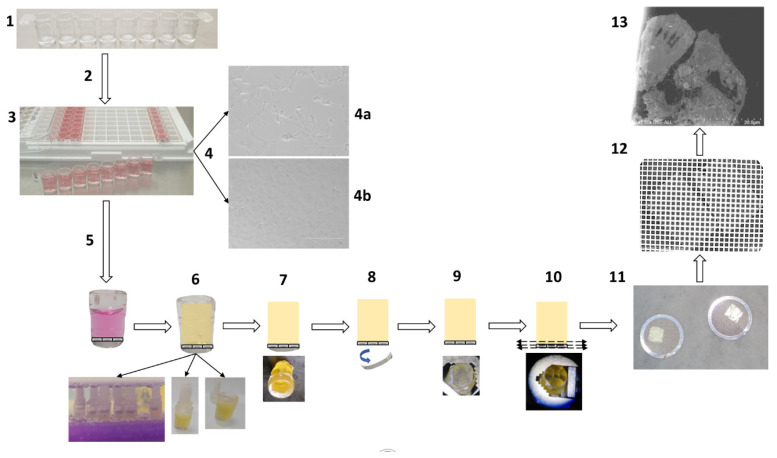
Schematic representation of the methodology for fast electron microscopic examination of a virus culture. (1) Removable single-break strips are UV sterilized and collagen-coated. (2) Cell culture with 200 µL suspensions of Vero E6 cells at 2 × 10^5^ cells/mL per well. (3) Cells are virus-inoculated with 50 µL of strains or clinical sample per well. (4) Cytopathic effect detection: cytopathic effect (4a) is monitored by bright-field light microscopy, in comparison with a negative control (4b). (5) Cell fixation: cell monolayers are fixed with 2.5% final glutaraldehyde. (6) In-well resin embedding in 4 h: washes, dehydration, resin substitution and wells closed by cap sealing for microwave polymerization. (7) Plastic pruning: resin blocks are manually trimmed with a razor blade. (8) Detachment of the cell monolayer: the plastic bottom of the well is detached by cold shock via immersion in liquid nitrogen for 20 s. (9) Resin block: the resin block containing the cell monolayer at one side is ready for ultramicrotomy. (10) Ultramicrotomy: the resin block is trimmed to a pyramid and ultra-thin sections are obtained. (11) Positioning on grids: ultra-thin sections are deposited on copper/rhodium grids. (12) Contrast and metal deposition: sections are contrasted with uranyl acetate and lead citrate and grids attached to a glass slide are platinum sputter-coated. (13) Electron microscopy: electron micrographs are obtained by SEM.

The total duration of a cell culture of SARS-CoV-2 virus-infected cell monolayers in a single-break strip microplate was three days. At each selected time-point (2, 3, 6, 12, 18, and 36 h post-infection, hpi), one well of the microplate was chemically fixed and stored at 4 °C. After collecting all time-points, the wells were directly resin-embedded. The total processing time for the resin embedding was 4 h, from the first fixative wash to cured resin blocks. For curing resin with microwaves, the culture wells needed to be closed, as wells were placed in water, and a 3D-printed cap was placed on top of the well to guarantee sealing. For resin block extraction from the plastic wells and ultramicrotomy in the plane of the cells, an extra day was needed. The size of the resin blocs obtained with our approach after extraction from the plastic of the wells was fully compatible with the size of the ultramicrotome specimen holder. Trimming was only performed to obtain a pyramid at the top. One final day was employed for ultra-thin section contrast with heavy metals and image acquisitions by SEM. Thus, from cell culture to observation, a total of 6.5 days were needed for a 36 h SARS-CoV-2 infectious cycle examination by EM. The entire process is described in Figure 1.

**Figure 2:**
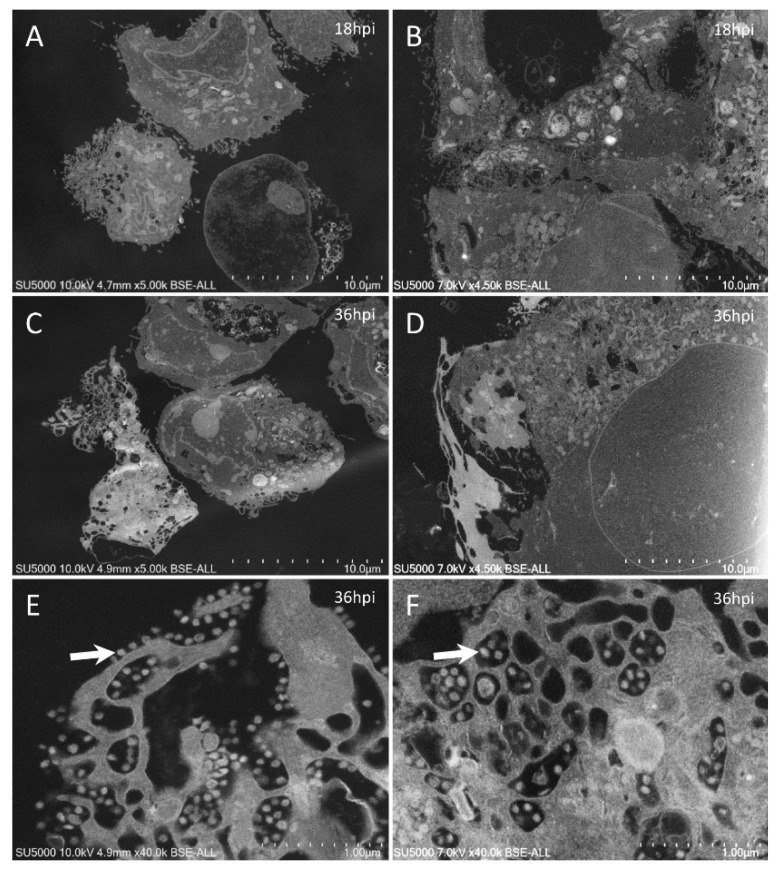
Ultra-thin sections of SARS-CoV-2-infected Vero E6 cells: comparison between images obtained by our classical methodology [9] (left column) and our new approach (right column). In sections obtained from a pellet of SARS-CoV-2-infected cells (**A**,**C**), cells were generally isolated and separated from each other, while in sections of SARS-CoV-2 monolayers (**B**,**D**), cells were usually clustered, contacting each other, and more spread out. With our new approach, SARS-CoV-2 particles (arrows) could be recognized (**F**), as was the case when using our classical methodology (**E**).

**Figure 3:**
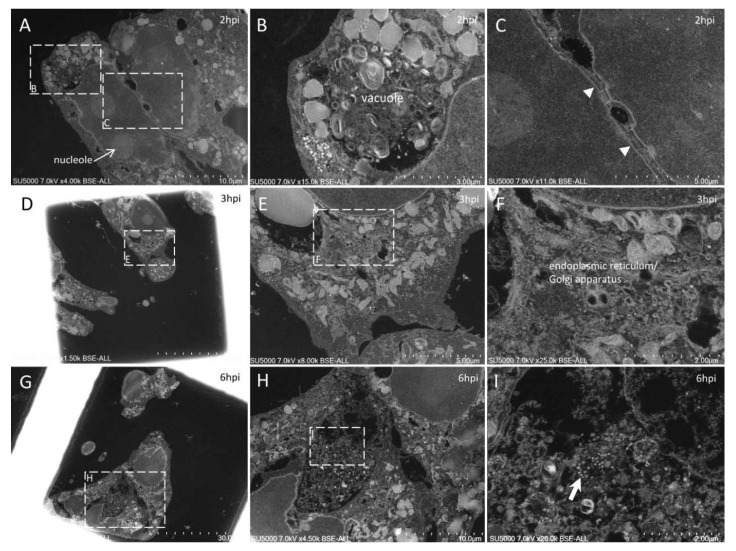
Ultra-thin sections of SARS-CoV-2-infected Vero E6 cells: 2–6 hpi. (**A**–**C**): 2 hpi. Two cells (**A**) with plasma membranes closely associated with a ‘Velcro’ organization (**C**; arrowheads). One of the two cells presents a large vacuole enriched in membranes and granules (**B**). (**D**–**I**): 3 hpi. One cell (**D**) presents the typical budding of the endoplasmic reticulum and/or Golgi apparatus compartments following SARS-CoV-2 infection (**E**,**F**). (**G**–**I**): 6 hpi. A region (**H**) located between intact cells and containing debris and mature SARS-CoV-2 particles (**I**; arrow).

**Figure 4:**
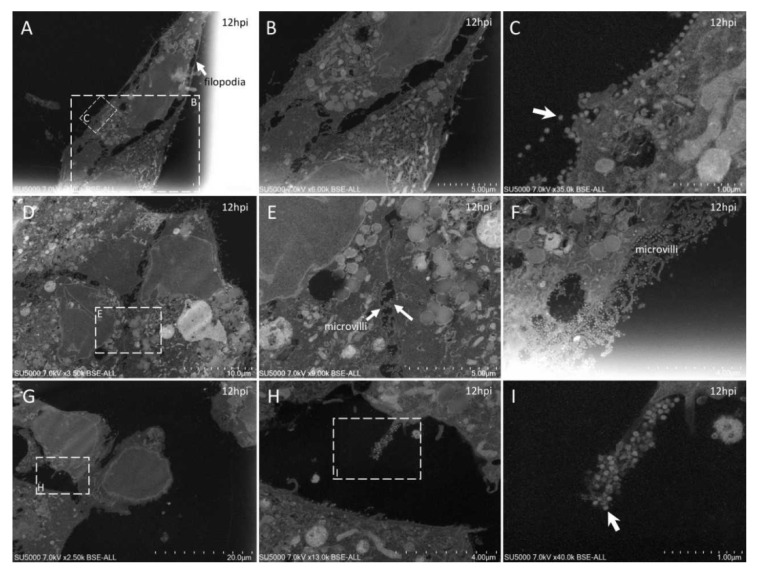
Ultra-thin sections of SARS-CoV-2-infected Vero E6 cells: 12 hpi. (**A**–**C**): Two neighboring cells, one with a long filopodia (**A**,**B**; arrow **A**) and one presenting extracellular SARS-CoV-2 particles at the plasma membrane (**C**; arrow). (**D**–**F**): SARS-CoV-2-enriched microvilli located between neighboring cells (**E**; arrows) or at the cell-free periphery (**F**). (**G**–**I**): One microvillus extremely enriched in SARS-CoV-2 virions (**I**; arrow).

**Figure 5:**
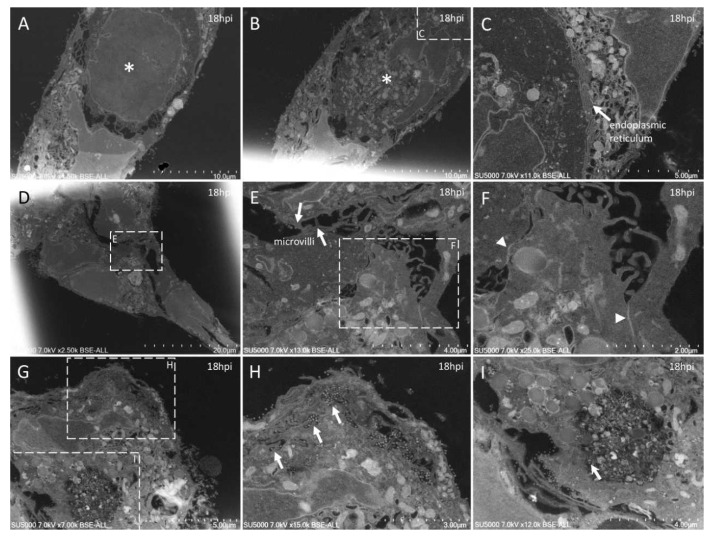
Ultra-thin sections of SARS-CoV-2-infected Vero E6 cells: 18 hpi. (**A**,**B**): Two serial sections of the same cell, with cuts at the level of its nucleus (*) at different heights along its depth. In (**C**), the nuclear membrane/endoplasmic reticulum presents a stacked organization (arrow). (**D**–**F**): Adjacent cells contacting each other through microvilli (**E**) or electron-dense regions resembling tight junctions (**F**; arrowheads). (**G**–**I**): SARS-CoV-2 particles (arrows) located inside a canalicular tubulo-vesicular system (**H**) as well as in a virus-morphogenesis vesicle (VMV; **I**) of the same cell.

**Figure 6:**
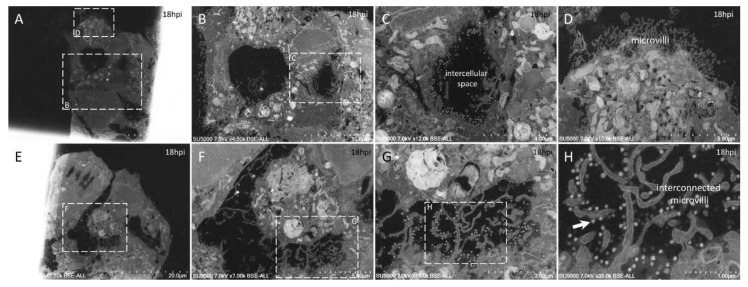
Ultra-thin sections of SARS-CoV-2-infected Vero E6 cells: 18 hpi. (**A**–**D**): Neighboring cells harboring extracellular SARS-CoV-2 particles at the level of the intercellular space (**C**) or at the level of peripheral microvilli (**D**). (**E**–**H**): A cellular cluster with two cells facing each other with intermingled microvilli enriched in SARS-CoV-2 particles (**H**; arrow).

**Figure 7:**
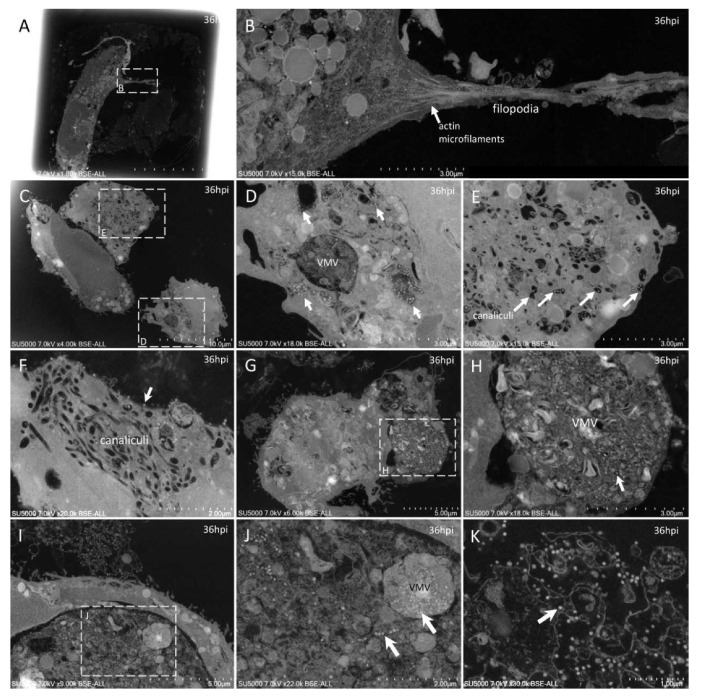
Ultra-thin sections of SARS-CoV-2-infected Vero E6 cells: 36 hpi. (**A**,**B**): A cell with a filopodia (**B**) containing actin microfilaments (arrow); SARS-CoV-2 virions were not detected inside such filopodia structures. (**C**–**F**): Cells with SARS-CoV-2 particles inside a virus-morphogenesis vesicle (VMV) or inside tubules/vesicles of the canaliculi system (**D**,**E**; arrows) or at the plasma membrane (**F**; arrow). (**G**,**H**): Large VMV containing SARS-CoV-2 virions (arrow) and membranes. (**I**–**K**): In adjacent cellular regions, SARS-CoV-2 particles (arrows) were found concentrated in a VMV, disseminated in a larger vacuole (**J**; arrows), or stitched extracellularly to the plasma membrane remnants of a lysed cell (**K**).

With our new methodology, we observed the previously described [9] round to hexagonal electron-dense SARS-CoV-2 particles, around 80 nm in diameter and more or less spiky, depending on their location (Figure 2). We also obtained previously described ultrastructural features of the SARS-CoV-2-infected cells [9,17,18,19,20,21,22]: neo-virions formed in the peri-nuclear region from budding of the endoplasmic reticulum–Golgi apparatus complex, with a formation of morphogenesis matrix vesicae, and new particles expelled from the cells through cell lysis or by fusion of virus-containing vacuoles with the cell plasma membrane (Figure 3, Figure 4, Figure 5, Figure 6 and Figure 7).

Our approach also showed new ultrastructural aspects of the SARS-CoV-2 infectious cycle in Vero E6 cells. Indeed, our ability to observe sections of intact cell monolayers instead of pelleted cells (Figure 2) showed the relationships between the cells, such as cell–cell contacts, and the characteristics of the cells at different levels along their cellular baso-apical axis. Cells could be seen touching each other (i) along large plasma membrane regions, with Velcro-like membrane appositions resembling adherent junctions (Figure 3A,C); (ii) at discrete locations with electron-dense contacts resembling tight junctions (Figure 5D–F); or (iii) by interconnecting microvilli (Figure 5E,F and Figure 6E–H). Large cellular protrusions could be seen (Figure 4A,B), potentially enwrapping neighboring cells (Figure 5A,B). We found filopodia containing actin microfilaments and presenting the characteristic V-shape at their base (Figure 7A,B). These filopodia were devoid of virion particles, either internally or on the surface. The filopodia were different from the numerous I-shaped microvilli (mean diameter, 90 ± 30 nm; n = 21), characteristic of epithelial Vero E6 cells (Figure 4D–I, Figure 5D–I and Figure 6). Microvilli were present in the basolateral cellular regions between more or less distant cells and in regions located more apically in the cells. Microvilli were enriched at their surface with mature SARS-CoV-2 virions, especially from 12 hpi. Strikingly, microvilli of neighboring cells could be seen intermingled, with an amount of virion particles proportional to the number of villi. While we had already observed the canalicular system of Vero E6 cells and its content in SARS-CoV-2 virions, this tubular and interconnected vacuolar system was more pronounced using our approach (Figure 5G,H and Figure 7C–F). We observed networks of tubules and compartments that ultimately connected to the external plasma membrane, mostly in apical cellular regions. The SARS-CoV-2-rich compartments connected to the canalicular system were smaller in diameter and less electron-dense when located closer to the plasma membrane, suggesting a progressive delivery of new virions as VMV matured and fused with the apical tubular system.

## 4. Discussion

We compared our new approach to our routine methodology that we had been using for studying the SARS-CoV-2 infectious cycle [9]. As for this former work, for imaging cell sections, we chose scanning electron microscopy for its strengths related to its speed of use and ability to rapidly illustrate cellular modifications following SARS-CoV-2 infection [9]. For higher resolution, transmission electron microscopy may also be used as a complementary imaging modality. We found our new method much faster for studying the ultrastructure of SARS-CoV-2-infected cells. Indeed, our method saved time; initially, at the stage of cell culture and, secondly, at the stage of electron microscopy. Regarding cell culture, three subcultures over a six-day period were needed to obtain a pellet of SARS-CoV-2-infected cells [9]. Here, with our new method that used breakable serology microplates, cell culture was shortened to the three days duration of the infectious cycle only. Regarding EM processing, we gained time first by a direct resin embedding of the cells in the culture wells, with no need for a special handling of a culture substrate. Secondly, the use of microwaves for accelerating EM processing is a well-known technique [23,24,25,26], and its use here proved very efficient, with EM processing duration shortened to only 4 h compared to the 4.5–5.5 days with standard non-microwave-assisted methods using heat-curing that we used previously [9]. Additional experimental time for ultramicrotomy, contrast, and EM observations depends on the number of wells/post-infection time-points that one wants to analyze. Here this duration was two days, but it may be shortened if less/shorter time-points need to be analyzed. One major methodological advantage of our approach is that one can monitor the virus-infected cell cultures by light microscopy and fix a cell monolayer to examine it once a cytopathic effect is noticed.

Our method also proved useful for observing in situ cellular ultrastructural features of SARS-CoV-2 infection in an intact cell monolayer. Indeed, one advantage of our method was that it enabled the observation of adherent undetached cells and thus the morphological relationships between cells. For example, SARS-CoV-2-infected Vero E6 cells were seen to be interconnected by microvilli (Figure 5E,F and Figure 6E–H). These microvilli were enriched with numerous SARS-CoV-2 particles (Figure 4F,I), as already described [9,18,20,22]. We showed here that not only the apical microvilli were enriched in SARS-CoV-2 particles, but also those that could be the intercellular baso-lateral microvilli. These microvilli thus may assist the dissemination of SARS-CoV-2 particles among cells in a monolayer or a tissue. Another example was the canalicular system and its content of SARS-CoV-2 virions, which was frequently observed in adherent Vero E6 cells (Figure 5G,H and Figure 7C–F), probably because cells can retain their conformation, compared to detached rounded cells in which intracellular compartments may change their spatial organization. Our images thus suggest a progressive delivery of new virions as VMV mature and fuse with the apical tubular system, with SARS-CoV-2-enriched compartments connected to the canalicular system. Accordingly, apical trafficking in Vero E6 cells and other SARS-CoV-2-infected epithelial cells may be of interest for pharmacological inhibition of SARS-CoV-2 dissemination to neighboring cells. As a complementary approach, our method provides ready-to-cut resin blocks with infected cell monolayers that may be very suitable for three-dimensional (3D) reconstructions [7] by serial-block face (SBF)/focused-ion-beam(FIB)-SEM experiments [27] or manually cut serial section ribbons by TEM/SEM (Figure 5A,B) [28].

## 5. Conclusions

Our innovative approach, compared with our routine EM workflow for studying virus-infected cells, provides a faster electron microscopic examination of virus-infected cells. As illustrated on SARS-CoV-2-infected cells, our method is also capable of revealing new ultrastructural findings, because it allows for the in situ visualization of cell monolayers. Finally, our approach offers perspectives for a faster characterization of unknown viruses and infectious cycles potentially involved in other epidemics or pandemics.

## Data Availability

Not applicable.

## Supplementary Materials

The following are available online at https://www.mdpi.com/article/10.3390/microorganisms9061194/s1, Table S1: Microwave resin embedding and polymerization settings (PELCO BioWave ProPlus).
